# Cooperative Interaction of *Janthinobacterium* sp. SLB01 and *Flavobacterium* sp. SLB02 in the Diseased Sponge *Lubomirskia* *baicalensis*

**DOI:** 10.3390/ijms21218128

**Published:** 2020-10-30

**Authors:** Ivan Petrushin, Sergei Belikov, Lubov Chernogor

**Affiliations:** 1 Limnological Institute, Siberian Branch of the Russian Academy of Sciences, Irkutsk 664033, Russia; sergeibelikov47@gmail.com (S.B.); lchernogor@mail.ru (L.C.); 2 Faculty of Business Communication and Informatics, Irkutsk State University, Irkutsk 664033, Russia

**Keywords:** symbiosis, opportunistic pathogens, *Janthinobacterium* sp., *Flavobacterium* sp., genomes, bioinformatics, floc formation, *Lubomirskia baicalensis*

## Abstract

Endemic freshwater sponges (demosponges, Lubomirskiidae) dominate in Lake Baikal, Central Siberia, Russia. These sponges are multicellular filter-feeding animals that represent a complex consortium of many species of eukaryotes and prokaryotes. In recent years, mass disease and death of *Lubomirskia baicalensis* has been a significant problem in Lake Baikal. The etiology and ecology of these events remain unknown. Bacteria from the families Flavobacteriaceae and Oxalobacteraceae dominate the microbiomes of diseased sponges. Both species are opportunistic pathogens common in freshwater ecosystems. The aim of our study was to analyze the genomes of strains *Janthinobacterium* sp. SLB01 and *Flavobacterium* sp. SLB02, isolated from diseased sponges to identify the reasons for their joint dominance. *Janthinobacterium* sp. SLB01 attacks other cells using a type VI secretion system and suppresses gram-positive bacteria with violacein, and regulates its own activity via quorum sensing. It produces floc and strong biofilm by exopolysaccharide biosynthesis and PEP-CTERM/XrtA protein expression. *Flavobacterium* sp. SLB02 utilizes the fragments of cell walls produced by polysaccharides. These two strains have a marked difference in carbohydrate acquisition. We described a possible means of joint occupation of the ecological niche in the freshwater sponge microbial community. This study expands the understanding of the symbiotic relationship of microorganisms with freshwater Baikal sponges.

## 1. Introduction

Endemic freshwater sponges (demosponges, Lubomirskiidae) dominate in Lake Baikal in the littoral zone. They cover up to 50% of available surfaces [1] and represent a complex consortium of many species of eukaryotes and prokaryotes, including diverse chlorophyll-containing microalgae [2,3,4]. The first appearance of anomalously pink-colored *Lubomirskia baicalensis* (Pallas, 1776) sponges was reported in 2011. In recent years, mass disease and death of freshwater sponges of the family Lubomirskiidae, have been noted as leading to significant negative changes in biota. Diseased and dying sponges have been observed in many areas of the lake [5,6,7]. The etiology and ecology of these events remains unknown.

Freshwater sponges are multicellular filter-feeding animals, meaning they filter large quantities of water and extract nutrients and other chemicals. In previous microbiome studies we reported a shift in microbial communities of the diseased Baikal sponges. This diversity shift was characterized by mass mortality of microalgae (Chlorophyta) and increased abundance of several opportunistic bacteria [7,8]. In healthy sponges, sample microalgae dominate and there is minimal abundance of bacteria [7]. The term “healthy sponge” represents samples of green sponges that have no visible external signs of disease such as ulceration, biofilm on their surface, darkening of tissue, or discoloration of the sponge body.

Bacteria in diseased sponges belong mainly to the phyla Bacteroidetes and Proteobacteria and are significantly more diverse at the family level. Among these, the families Flavobacteriaceae and Oxalobacteraceae are dominant. We experimentally infected the healthy cell culture of primmorphs with cell suspension from diseased sponges and then observed the increase in the relative abundance of Flavobacteriaceae and Oxalobacteraceae in these cell cultures [8]. Then we isolated and cultivated separately and performed whole-genome sequencing of two dominating strains named *Janthinobacterium* sp. SLB01 (Proteobacteria, Betaproteobacteria, Burkholderiales, Oxalobacteraceae, *Janthinobacterium*) and Flavobacterium sp. SLB02 (Bacteroidetes, Flavobacteriia, Flavobacteriales, Flavobacteriaceae, *Flavobacterium*) respectively [9,10].

Most *Janthinobacterium* bacteria can produce violacein, a known metabolite of *Janthinobacterium lividum* which has antibacterial activity [11]. Violacein biosynthesis is associated with cell density and controlled by quorum sensing [12,13]. Three key genes that encode proteins and that are associated with quorum sensing, are the CAI-1/LAI-1 autoinducer synthase, two-component histidine sensor kinase and a two-component response regulator. These were discovered in a previous study of the closer species *Janthinobacterium* sp. HH01 [12].

As reported previously, in the stationary phase, *Janthinobacterium lividum* forms a strong biofilm that is rich in exopolysaccharides [13]. As a representative of active sludge bacteria, some *Janthinobacterium* can produce granules or floc [14]. The crucial factor of floc formation (with exopolysaccharides (EPS) synthesis) is the expression and further post-translational processing of specific PEP-CTERM/XtrA proteins with their PrsK-PrsR regulatory system [15]. PEP-CTERM-containing proteins generally contain an N-terminal signal peptide and exhibit high diversity and little homology to known proteins. These proteins add proteinaceous components when the cell produces EPS. All bacteria with PEP-CTERM have both an outer membrane and EPS production genes [16].

The members of the genus *Flavobacterium*, which belong to the phylum Bacteroidetes, are typical commensal bacteria of soil, saline, and freshwater ecosystems that can be opportunistic pathogens [17,18]. Genomes of terrestrial isolates are almost two times larger than those of aquatic isolates [19]. Terrestrial forms of *Flavobacterium* are very diverse and have a large number of carbohydrates metabolism genes. The aquatic forms have a high percentage of protein and peptide utilization genes.

In a number of previous works, researchers have shown that some species of *Flavobacterium* contain proteolytic and collagenolytic enzymes [20,21]. These bacteria regulate a diverse array of activities, including symbiosis, antibiotic production, motility, virulence, and biofilm formation [22,23,24].

Here we present full genome sequence analysis of *Janthinobacterium* sp. SLB01 and *Flavobacterium* sp. SLB02, which were isolated from diseased sponges, in order to understand their joint action in the diseased sponges. Obtaining and comparing genomes is an important step towards understanding the relationships in the microbial community during the mass mortality of freshwater Baikal sponges.

## 2. Results

### 2.1. Cultivation Strains, Genome Assembly, Scaffolding and Features

Strains were gram-stain-negative and aerobic. Colonies of *Janthinobacterium* sp. SLB01 are violet pigmented single rods, 0.2–0.3 µm in diameter and 1.0–2.0 µm long, with gliding motility. Yellow pigmented colonies of the *Flavobacterium* sp. SLB02 strain are rod-shaped bacteria 0.75–1.0 µm in width and 3–5 µm in length. Bacterial DNA was extracted and sequenced using the Illumina MiSeq platform as described in [9,10].

After draft assembly with SPAdes [25], we constructed the reference-assisted scaffolding using Ragout [26]. Genome completeness analysis with benchmarking universal single-copy orthologs (BUSCO, [27]) showed results of: *Flavobacterium* sp. SLB02 96.2% complete, 1.1% fragmented, and 2.7% missing BUSCOs; *Janthinobacterium* sp. SLB01 98.2% complete, not fragmented, and 1.8% missing BUSCOs. 

Genomes were released in NCBI for further study and annotation. The final genome assembly statistics of: raw reads count, genome size, number of genes, pseudogenes, protein-coding sequences, tRNA noncoding RNA, and references to genome reports are presented in Table 1.

### 2.2. Phylogenetic Relationship with Closer Species

**Figure 1:**
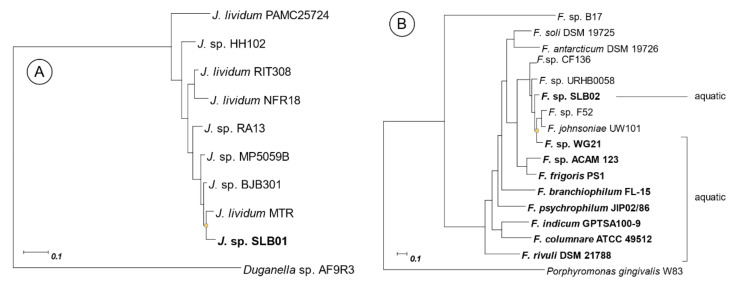
Phylogenetic tree of *Janthinobacterium* sp. SLB01 (**a**) and *Flavobacterium* sp. SLB02 (**b**) with closely related species. Trees are built based on approximately 400 universal marker genes by PhyloPhlAn (a maximum-likelihood method). GenBank accession numbers are given in Tables S1a and S1b. Strains of *Flavobacterium* with an aquatic niche are highlighted with a bold font.

To compare genomic features of *Janthinobacterium* sp. SLB01 and *Flavobacterium* sp. SLB02 with closer species we built phylogenetic trees for some of these species (Figure 1). The strains list for *Janthinobacterium* was taken from [28], in which the quorum sensing system for *Janthinobacterium* was described. The strain list for *Flavobacterium* was taken from a study of terrestrial and aquatic niches of this species [19]. Genome properties of each strain in this study are presented in Tables S1a and S1b. 

### 2.3. Violacein Synthesis by Janthinobacterium sp. SLB01

**Figure 2:**
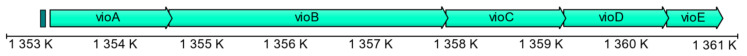
Diagram of the violacein production loci in the *Janthinobacterium* sp. SLB01 genome. Genes are displayed with arrows. A conserved sequence motif within the promoter region is displayed with the gray bar.

The strain *Janthinobacterium* sp. SLB01 was able to produce violacein. Its genome contains required violacein synthesis operon vioABCDE. The locus structure is presented in Figure 2 and gene coordinates and locus names are presented in Table S2.

### 2.4. Type VI Secretion System Genes Identification

**Figure 3:**
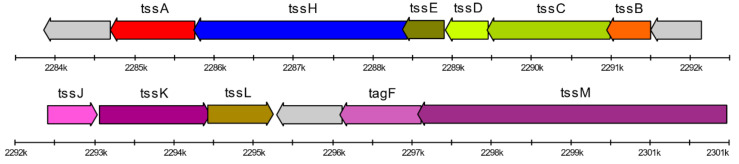
Schematic diagram of the genetic organization of the type VI secretion system main gene cluster in the *Janthinobacterium* sp. SLB01 genome.

The genome of *Janthinobacterium* sp. SLB01 contains all three categories of the genes that are required for the function of the type VI secretion system. These genes are allocated through the genome by 10 clusters, the largest of which containing most of the genes shown in Figure 3. We also analyzed other closer strains of *Janthinobacterium* (Figure 1A and Table S1a). All of these (with the exception of *Janthinobacterium* sp. HH102) do not have all of the required genes for T6SS function. The name, locus, localization, and annotation of each gene are presented in the Supplementary Materials (Table S3).

### 2.5. Quorum Sensing in Janthinobacterium sp. SLB01

We found genes associated with the quorum sensing in the *Janthinobacterium* sp. SLB01 genome. The three key genes were the synthesis of the CAI-1/LAI-1 autoinducer synthase, a two-component histidine sensor kinase, and a two-component response regulator. The localization and homology percentage of these genes are presented in Table 2. The violacein operon and quorum sensing (QS) cluster have a common regulatory motif that would be located before the QS regulated genes as described in [28]. This motif has the pattern TTGA(N_6/7_) TCAA and is located in the intergenic region before the locus F3B38_RS23480 (HAMP domain-containing histidine kinase) with the motif sequence “TTGACGTATATCAA” and before the locus F3B38_RS17235 (begin of violacein operon) with the motif sequence “TTGATATTTATCAA.”

### 2.6. Floc Formation by Janthinobacterium sp. SLB01

**Figure 4:**
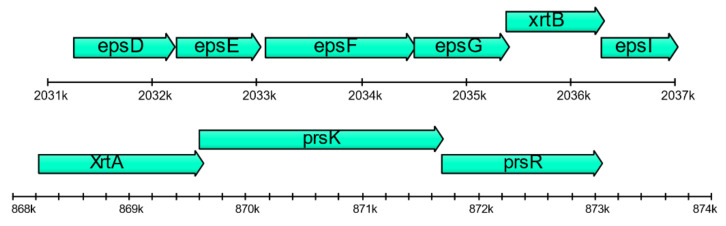
Schematic diagram of the genetic organization of *Janthinobacterium* sp. SLB01 gene clusters required for floc formation: exopolysaccharides (EPS) synthesis, PEP-CTERM, and exosortase. Genes are indicated by arrows and the direction of the arrows represents the direction of transcription of the genes in the genome.

In the stationary phase, *Janthinobacterium* sp. SLB01 formed a strong biofilm that was rich in exopolysaccharides (EPS). Its genome has large (F3B38_RS08235–F3B38_RS08375) and small (F3B38_RS15000–F3B38_RS15020) gene clusters and containing genes of synthesis and export polysaccharides (epsDEFGI). These genes may also be involved in extracellular polysaccharide biosynthesis (see Figure 4). Secretion of EPS and expression of PEP-CTERM proteins and exosortase can form the floc [15]. Gene clusters include the TIGR03013 family PEP-CTERM/XrtA system glycosyltransferase (previously called EpsH), PEP-CTERM system histidine kinase PrsK, and PEP-CTERM-box response regulator transcription factor PrsR. Genome analysis of *Janthinobacterium* sp. SLB01 revealed all of the required gene clusters for floc formation. In total, at least 27 genes encoding typical PEP-CTERM proteins were identified in the *Janthinobacterium* sp. SLB01 strain. In another study of floc formation, two glutamine-dependent asparagine synthases (asnB or asnH) were required [29] and also have orthologs in the *Janthinobacterium* sp. SLB01 genome (F3B38_RS08405).

**Figure 5:**
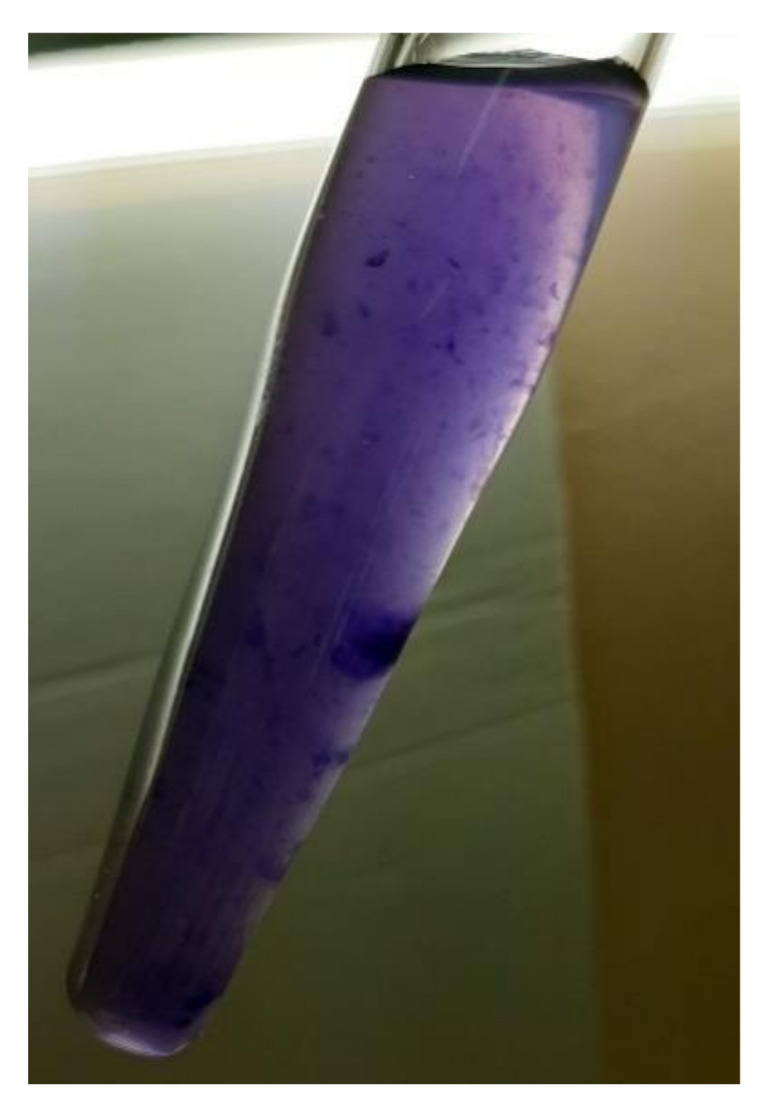
Visual observance of floc formation and violacein synthesis by *Janthinobacterium* sp. SLB01.

Floc formation and violacein pigment are visually observed in the cell culture of *Janthinobacterium* sp. SLB01 (see Figure 5).

### 2.7. Comparative Genomics of Janthinobacterium Species

To compare genomic features, we checked if closely related species of *Janthinobacterium* (listed in Table S1a, Figure 1A) have homologs for the genes of interest described above (see Section 2.3, Section 2.4, Section 2.5 and Section 2.6). The presence or absence of each gene is shown in Table S4. The search for homologous genes was performed using Roary core/pan-genome software [30] followed by manual verification.

### 2.8. Polysaccharides Utilization Loci Analysis in Flavobacterium sp. SLB02

A total of 45 polysaccharide utilization loci (PULs) were predicted in the *Flavobacterium* sp. SLB02 genome. These were annotated and are available online in PULDB http://www.cazy.org/PULDB/index.php?sp_name=Flavobacterium+sp.+SLB02 [31]. Each PUL consists of SusC/D marker genes with various combinations of glycoside hydrolases, carbohydrate-binding modules, carbohydrate esterases, polysaccharide lyases, extracytoplasmic function σ-factor, peptidases and transporters (all definitions are described at http://www.cazy.org/PULDB/tags.html).

**Figure 6:**
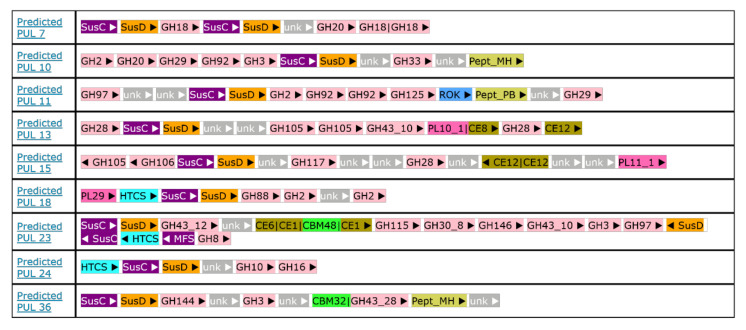
Polysaccharide utilization loci (PULs) of the *Flavobacterium* sp. SLB02 genome that have homologous PULs in the *Flavobacterium johnsoniae* UW101 genome.

Genomic comparisons showed that homologous loci to these PULs occur in other Bacteroidetes members, some of which experimental data about utilized polysaccharides exists. PULs with numbers 4, 7, 10, 11, 13, 15, 18, 23, 24 and 36 have strong homology (according to PULDB reports) with *Flavobacterium johnsoniae* UW101. The map of each of these loci is presented in Figure 6. 

### 2.9. Carbon Sources Metabolism

Genome analysis undertaken with SEED shows that *Janthinobacterium* sp. SLB01 and *Flavobacterium* sp. SLB02 use different carbohydrates as carbon sources. The SEED pipeline groups the sets of related functional roles as “subsystems” which are defined as a set of functional roles that an annotator has decided should be thought of as related. As previously noted, *Flavobacterium* sp. SLB02 can utilize polysaccharides, but *Janthinobacterium* sp. SLB01 mostly cannot (it has genes for chitin degradation only). We analyzed the feature counts for the carbohydrate metabolism subsystem and found that the composition of carbon acquisition genes is different for a number of enzymes. Table 3 lists the pathways (or subsystems in SEED) with significant (present or absent) differences. The full list of subsystems is presented in Table S5.

## 3. Discussion

### 3.1. The Role of Each Species in the Joint Action

Bacteria of *Janthinobacterium* sp. SLB01 can colonize the space and suppress the other bacteria (especially gram-positive bacteria) with violacein. This pigment production was observed in cell culture (Figure 5) and all required genes (operon VioABCDE) are present in its genome. Violacein is also associated with quorum sensing (QS) and biofilm formation [13]. A cluster of three key genes associated with quorum sensing is a synthesis of the CAI-1/LAI-1 autoinducer synthase, a two-component histidine sensor kinase, and a two-component response regulator. Few studies exist that describe quorum sensing for the *Janthinobacterium* family. We used the description of the QS system for the *Janthinobacterium* sp. HH01 strain [12,28] and found genes homologous to all of the required genes in the *Janthinobacterium* sp. SLB01 genome. Localization, annotation, and identity percentage of these genes are presented in Table 2.

When cultivating *Janthinobacterium* sp. SLB01, we experimentally observed biofilms and floc formation in the cell cultures of primmorphs of *L. baicalensis* (unpublished data). Exopolysaccharides (EPS) are the main component of biofilm produced by the species of the Oxalobacteraceae and Flavobacteriaceae families [13,32,33]. *Janthinobacteria* usually form biofilms on eukaryotic hosts and are known to synthesize antibacterial and antifungal connections [12]. These bacteria can also produce the floc and strong biofilm in the stationary phase. This process requires exopolysaccharide biosynthesis. However, a recent study it was clarified that both widespread PEP-CTERM proteins and exopolysaccharides are required for floc formation [15]. For all of the gene clusters required for floc formation, we found the respective homologs in the *Janthinobacterium* sp. SLB01 genome (Figure 4). Floc formation can negatively affect breathing, nutrient acquisition, and waste product removal of the host (sponge *L. baicalensis*) due to clogging of the pores. These negative effects of biofouling on the functioning of the filter-feeding sponge *Halisarca caerulea* has been studied [34]. 

One essential strategy of gram-negative bacteria is the secretion of virulence factors through the cell membranes of the victim to achieve a potential target. *Janthinobacterium* sp. SLB01 attacks the eukaryotic and bacterial cells via T6SS, and then takes the released nutrients after cell lysis. Its genome contains all three categories of genes that are required for the function of the type VI secretion system (T6SS) [35,36]. The first category consists of three genes encoding proteins, which form a membrane-associated complex (two for the inner membrane (TssL, TssM) and one for the outer membrane (TssJ)). The genes of the second category encode proteins similar to the bacteriophage sheath (TssD or Hcp, VgrG, TssB, TssC, TssE). The last genes encoding the proteins control Hcp tube formation (VgrG, TssA, TssF, TssG, TssK). It should be noted, that the activity of T6SS is limited due to the length of the “spike” formed with VgrG and Hcp proteins. Cell walls (which should remain after cell lysis) block further T6SS function, but some other bacteria can utilize them.

*Flavobacterium* sp. SLB02 is a the gram-negative, opportunistic bacterium of the phylum Bacteroidetes. Like many Bacteroidetes, it has specific polysaccharide utilization loci (PULs). After release in GenBank, its genome was analyzed by PULDB [31]. A large number of annotated PULs (in PULDB) allowed their composition to be analyzed and a comparison made with other genomes of the Flavobacterium family. We predicted some of the polysaccharides, that *Flavobacterium* sp. SLB02is able to digest by comparing annotated PULs with the literature-derived data stored in PULDB [31]. Ten of the 45 PULs found in the *Flavobacterium* sp. SLB02 genome have strong homology (according to PULDB reports) with *Flavobacterium johnsoniae* UW101 (see Figure 6). 

According to a previous study [37,38], these PULs enable the digestion of a wide range of polysaccharides, which was predicted based on the genome analysis and confirmed experimentally. Digestible polysaccharides include cellulose, starch, α-glucan, and hemicelluloses (xylans, mannans, and xyloglucans). Predicted cell surface proteins related to *Bacteroides thetaiotaomicron* SusC and SusD, which are likely involved in the binding of oligosaccharides and in transport across the outer membrane, were also identified. Utilization of cell wall fragments clears the surrounding area, including T6SS activity of *Janthinobacterium* sp. SLB01.

The two investigated strains have differences in carbon acquisition. We analyzed the feature counts for carbohydrates and other carbon source metabolism subsystems using RAST SEED [39]. We found that the ratio of carbon acquisition genes is different for a large number of enzymes. The pathways (or subsystems in SEED) with significant (when one strain has genes, but the second does not) differences are listed in Table 3. This list contains almost half of the total number of pathways. We suggest that *Janthinobacterium* sp. SLB01 and *Flavobacterium* sp. SLB02 have little or no competition for carbon sources such as simple sugars, organic acids, and polysaccharides.

A large number of separate studies exist for each of the investigated strains in a freshwater niche. *Janthinobacterium* can live in cold conditions [40,41,42,43]; and *Flavobacterium* is a well-known fish pathogen [32] and a component of activate sludge [44,45,46]. Comparison with closer species (Table S1a) shows significant differences in the presence of genes of virulence in *Janthinobacterium* (see details on Table S4). Only two of these (SLB01 and HH102) have all of the genes required for T6SS function.

The above discussion shows that the two investigated strains are related in different phyla and have significantly different lifestyles in terms of virulence mechanism and, digested polysaccharides and carbohydrates (feeding). However, it is likely they act together hence explaining their dominance in the microbial community. Obtaining and comparing genomes is an important step towards understanding the relationships in the microbial community during the mass mortality of freshwater Baikal sponges.

### 3.2. Probable Scenario of Strains Interaction

We suggest the following probable scenario and sequence of events of the interactions of the studied bacteria in the microbiome of a diseased sponge. The *Janthinobacterium* sp. SLB01 possesses a flagellum and can lead a free-living planktonic lifestyle. However, in some cases, this bacterium can produce the floc, penetrating into the aquifer system of the sponges. When reaching a certain cell density, the quorum sensing (QS) changes the gene expression of *Janthinobacteria*, which activates the violacein synthesis. Violacein inhibits the growth of gram-positive bacteria (including symbiotic *Actinobacteria*), which changes in the microbiome and partially weakens the defense mechanism of the sponges.

The QS system also induces the activity of the type VI secretion system in *Janthinobacteria*, promoting the introduction of various effector proteins, including lytic enzymes, into neighboring eukaryotic or bacterial cells. Cell lysis releases the nutrients necessary for bacterial growth, in particular, *Janthinobacteria* and *Flavobacteria*. However, the type VI secretion system has limitations because it is able to transport effector proteins only to neighboring cells. Cell walls of killed bacteria or green microalgae form a barrier around the cells of *Janthinobacteria*, restraining their proliferation in the tissues of the sponges. 

These cell walls consist of soluble proteins, carbohydrates and polysaccharides. Some species can help *Janthinobacteria* to dissolve or utilize cell walls and remove the barrier that inhibits reproduction. The helper bacteria may not have the ability to kill or inhibit the growth of other living bacteria but must produce enzymes to hydrolyze high molecular weight proteins and polysaccharides efficiently.

*Flavobacterium* sp. SLB02 is probably such an effective helper that it contributes to the development of the sponges’ disease, death, and their complete disappearance. This strain was isolated from the aquatic environment of the sponge, but can be placed in the terrestrial clade of *Flavobacteria* due to its large genome size and the presence of a large number of polysaccharides utilization loci (PULs). It can hydrolyze a wide range of polysaccharides, including peptidoglycan, solid chitin, and cellulose.

*L. baicalensis* has a close symbiosis with green microalgae, which are a source of additional nutrition in the form of glycerates [4]. These microalgae can be an object of the joint action of two bacteria and eating algae can weaken the sponges due to starvation. 

The proposed scenario is speculative and does not take into account many details of the disease due to the complexity of the object of study. However, in our opinion, some aspects can be verified and confirmed in further laboratory experiments on the culture of sponge symbiotic green algae infected with strains SLB01 and SLB02 under separate or combined exposure.

## 4. Materials and Methods

### 4.1. Bacterial Strains and Growth Media

In this study, two strains were isolated from samples of the diseased sponge *L. baicalensis* (collected in Lake Baikal, Central Siberia, Russia). The cell suspension was isolated from these diseased sponges. The bacterial biomass was cultured on nutrient media with R2A (0.05% yeast extract, 0.05% tryptone, 0.05% casamino acids, 0.05% dextrose, 0.05% soluble starch, 0.03% sodium pyruvate, 1.7 mM K_2_HPO_4_, 0.2 mM MgSO_4_, final pH 7.2 adjusted with crystalline K_2_HPO_4_ or KH_2_PO_4_) agar plates (Merck KGaA, Darmstadt, Germany) at 15 °C, at pH 6.0–7.5, with the removal of fast-growing colonies. Then, in a series of successive passages, individual colonies of microorganisms were obtained. Cell morphology was determined by light microscopy Axio Imager Z2 microscope (Zeiss, Oberkochen, Germany) equipped with fluorescence optics (self-regulating, blue HBO 100 filter, 358/493 nm excitation, 463/520 nm emission). The isolates of bacteria were stained with a NucBlue Live ReadyProbes reagent (Thermo Fisher Scientific Inc., Waltham, MA, USA). The isolates were preserved as a 20% (*v/v*) glycerol suspension at 70 °C. Violet pigmented *Janthinobacterium* sp. SLB01 and yellow pigmented *Flavobacterium* sp. SLB02 strains were isolated on Luria–Bertani (LB) broth medium agar plates (diluted 1/10, temperature 15 °C).

### 4.2. Genome Assembly, Annotation and Phylogenetic Relationship

Draft assembly was performed using SPAdes version 3.11.0 [25] with default settings, raw reads error correction, and filtering with a built-in BayesHammer module (quality threshold 98%). The resulting contigs were ordered with Ragout version 2.2 with default settings (https://github.com/fenderglass/Ragout) [26].

Genome completeness analysis was conducted using BUSCO v. 3.1.0 and default settings with datasets “proteobacteria_odb9” with 221 BUSCOs for *Janthinobacterium* sp. SLB01 and “bacteroidetes_odb9” with 443 BUSCOs for *Flavobacterium* sp. SLB02 [27].

Annotation was performed with NCBI Prokaryotic Genome Annotation Pipeline (PGAP) [47], some genes were re-annotated with BLAST against the Swiss-Prot database and protein sequences of closely related species. 

A phylogenetic tree for each set of strains (Tables S1a and S1b) was constructed using PhyloPhlAn based on concatenated alignments of up to 400 conserved proteins [48] using “supermatrix_aa” and low diversity mode with the “phylophlan” database. We describe specific options of each tool embedded in PhyloPhlan 3.0 in Table S1c. Other settings were set to defaults.

### 4.3. In Silico Analysis of Type VI Secretion System Loci

A genome-wide analysis was performed in this study to reveal the veil of T6SS in the *Janthinobacterium* sp. SLB01. The components and location of T6SS homologs in *Janthinobacterium* sp. SLB01 was determined by SecReT6 (http://db-mml.sjtu.edu.cn/SecReT6/, mode T6SS-HMMER) integrated database with default settings [49].

### 4.4. Genome Subsystems and Comparative Genomics

We analyzed the subsystems of *Janthinobacterium* sp. SLB01 and *Flavobacterium* sp. SLB02 by RAST SEED (http://rast.nmpdr.org/) with default settings [39]. Detailed reports are available upon request.

Violacein synthesis genes (VioABCDE operon) were annotated by NCBI PGAP and verified by BLAST against protein sequences from the Swiss-Prot database.

Genes encoding PEP-CTERM proteins were partially annotated by NCBI PGAP. We used the gene list from the floc formation study [15] and found required homologs manually using UGENE [50]. Homologous gene searches were undertaken with Roary core/pan-genome software [30] with default settings and followed by manual checking.

### 4.5. Polysaccharides Utilization Loci Analysis

The genome of *Flavobacterium* sp. SLB02 was released in NCBI in 2019. Maintainers of PULDB [31] added this genome to the database and analyzed it by using a fully automated pipeline for PUL prediction using genomic context and domain annotation [51]. A detailed report is available at http://www.cazy.org/PULDB/index.php?sp_name=Flavobacterium+sp.+SLB02.

To compare 45 detected PULs in the *Flavobacterium* sp. SLB02 genome with literature-derived data we performed a similarity search using the PULDB built-in function. Hits with the highest score were then analyzed manually.

## Figures and Tables

**Table 1:** Raw reads and genome feature statistics of bacterial strains in this study.

Property	*Janthinobacterium* sp. SLB01	*Flavobacterium* sp. SLB02
Raw reads	12,099,942 *	17,921,744 *
GenBank accession number	VZAB00000000 **	CP045928 ***
Genome size, bp	6,467,981	6,363,829
Number of contigs	2	1
GC content	62.63%	35.50%
Number of genes	6023	4964
Protein-coding sequences	5863	4901
tRNAs	65	56
Noncoding RNAs	4	3
Pseudogenes	78	73
Reference to genome report	[9]	[10]

* The sequence library was generated from DNA using an Illumina Nextera XT DNA sample preparation kit. Whole-genome sequencing was performed using the Illumina MiSeq platform with paired-end chemistry (2 × 250 bp). ** Reference genome: *Janthinobacterium* sp. strain LM6 chromosome (GenBank accession number CP019510). *** Reference genomes: *Flavobacterium* sp. strain KBS0721 chromosome (GenBank accession no. CP042170) and the *Flavobacterium piscis* strain CCUG 60099 whole-genome sequence (GenBank accession no. MUHC01000000).

**Table 2:** Quorum sensing gene description in the *Janthinobacterium* sp. SLB01 genome.

*Janthinobacterium sp.* SLB01		*Janthinobacterium sp.* HH01		
Locus Tag	Annotation	Locus Tag	% Ident **	% Simi-larity **
F3B38_RS23475	quorum-sensing autoinducer synthase	Jab_2c24330 *	68.6	81.0
F3B38_RS23480	HAMP domain-containing histidine kinase	Jab_2c24340	60.6	73.1
F3B38_RS23485	response regulator	Jab_2c24350	68.2	79.8

* CAI-1/LAI-1 autoinducer synthase for the first time identified for *Janthinobacterium* specie [12]. ** Identity: the extent to which two sequences have the same residues at the same positions in alignment (in percent). The similarity between two sequences can be expressed as percent sequence identity and/or percent positive (similar) substitutions.

**Table 3:** Carbon sources and metabolism subsystem differences in the *Janthinobacterium* sp. SLB01 and *Flavobacterium* sp. SLB02 genomes.

Carbon Source Group	Subsystem * Name	SLB01 **	SLB02 ***
Central carbohydrate Metabolism	TCA Cycle	15	0
	Pentose phosphate pathway	0	9
Di- and oligosaccharides	Sucrose utilization	0	2
Organic acids	Methylcitrate cycle	7	0
	Propionate-CoA to succinate module	6	0
	Lactose and galactose uptake and utilization	0	8
Fermentation	Mixed acid	0	7
Polysaccharides	Glycogen metabolism	0	4
Monosaccharides	2-Ketogluconate utilization	4	0
	L-Arabinose utilization	0	9

* A subsystem is a set of functional roles that an annotator has decided should be thought of as related. ** Subsystem feature counts for *Janthinobacterium* sp. SLB01. *** Subsystem feature counts for *Flavobacterium* sp. SLB02.

## Supplementary Materials

Supplementary materials can be found at https://www.mdpi.com/1422-0067/21/21/8128/s1.

## Abbreviations

BUSCO	Benchmarking universal single-copy orthologs
CTERM	C-terminal
EPS	Exopolysaccharides
PGAP	Prokaryotic genome annotation pipeline
PEP	Pro-Glu-Pro
PUL	Polysaccharides utilization loci
T6SS	Type VI secretion system

## References

[1] Pile A.J., Patterson M.R., Savarese M., Chernykh V.I., Fialkov V.A. (1997). Trophic effects of sponge feeding within Lake Baikal’s littoral zone. 2. Sponge abundance, diet, feeding efficiency, and carbon flux. Limnol. Oceanogr..

[2] Jensen K.S., Pedersen M.F. (1994). Photosynthesis by symbiotic algae in the freshwater sponge, Spongilla lacustris. Limnol. Oceanogr..

[3] Chernogor L., Denikina N., Kondratov I., Solovarov I., Khanaev I., Belikov S., Ehrlich H. (2013). Isolation and identification of the microalgal symbiont from primmorphs of the endemic freshwater sponge Lubomirskia baicalensis (Lubomirskiidae, Porifera). Eur. J. Phycol..

[4] Bil K., Titlyanov E., Berner T., Fomina I., Muscatine L. (1999). Some aspects of the physiology and biochemistry of Lubomirska baikalensis, a sponge from Lake Baikal containing symbiotic algae. Symbiosis.

[5] Kravtsova L.S., Izhboldina L.A., Khanaev I.V., Pomazkina G.V., Rodionova E.V., Domysheva V.M., Sakirko M.V., Tomberg I.V., Kostornova T.Y., Kravchenko O.S. (2014). Nearshore benthic blooms of filamentous green algae in Lake Baikal. J. Gt. Lakes Res..

[6] Khanaev I.V., Kravtsova L.S., Maikova O.O., Bukshuk N.A., Sakirko M.V., Kulakova N.V., Butina T.V., Nebesnykh I.A., Belikov S.I. (2018). Current state of the sponge fauna (Porifera: Lubomirskiidae) of Lake Baikal: Sponge disease and the problem of conservation of diversity. J. Gt. Lakes Res..

[7] Belikov S., Belkova N., Butina T., Chernogor L., Van Kley A.M., Nalian A., Rorex C., Khanaev I., Maikova O., Feranchuk S. (2019). Diversity and shifts of the bacterial community associated with Baikal sponge mass mortalities. PLoS ONE.

[8] Chernogor L., Klimenko E., Khanaev I., Belikov S. (2020). Microbiome analysis of healthy and diseased sponges Lubomirskia baicalensis by using cell cultures of primmorphs. PeerJ.

[9] Petrushin I.S., Belikov S.I., Chernogor L.I. (2019). Draft Genome Sequence of Janthinobacterium sp. Strain SLB01, Isolated from the Diseased Sponge Lubomirskia baicalensis. Microbiol. Resour. Announc..

[10] Petrushin I.S., Belikov S.I., Chernogor L.I. (2020). Draft Genome Sequence of Flavobacterium sp. Strain SLB02, Isolated from the Diseased Sponge Lubomirskia baicalensis. Microbiol. Resour. Announc..

[11] Durán N., Justo G.Z., Ferreira C.V., Melo P.S., Cordi L., Martins D. (2007). Violacein: Properties and biological activities. Biotechnol. Appl. Biochem..

[12] Hornung C., Poehlein A., Haack F.S., Schmidt M., Dierking K., Pohlen A., Schulenburg H., Blokesch M., Plener L., Jung K. (2013). The Janthinobacterium sp. HH01 Genome Encodes a Homologue of the V. cholerae CqsA and L. pneumophila LqsA Autoinducer Synthases. PLoS ONE.

[13] Pantanella F., Berlutti F., Passariello C., Sarli S., Morea C., Schippa S. (2007). Violacein and biofilm production in Janthinobacterium lividum. J. Appl. Microbiol..

[14] Aqeel H., Basuvaraj M., Hall M., Neufeld J.D., Liss S.N. (2016). Microbial dynamics and properties of aerobic granules developed in a laboratory-scale sequencing batch reactor with an intermediate filamentous bulking stage. Appl. Microbiol. Biotechnol..

[15] Gao N., Xia M., Dai J., Yu D., An W., Li S., Liu S., He P., Zhang L., Wu Z. (2018). Both widespread PEP-CTERM proteins and exopolysaccharides are required for floc formation of Zoogloea resiniphila and other activated sludge bacteria. Environ. Microbiol..

[16] Haft D.H., Paulsen I.T., Ward N., Selengut J.D. (2006). Exopolysaccharide-associated protein sorting in environmental organisms: The PEP-CTERM/SpsH system. Application of a novel phylogenetic profiling heuristic. BMC Biol..

[17] Chen S., Blom J., Loch T.P., Faisal M., Walker E.D. (2017). The emerging fish pathogen Flavobacterium spartansii isolated from Chinook salmon: Comparative genome analysis and molecular manipulation. Front. Microbiol..

[18] Kinnula H., Mappes J., Sundberg L.R. (2017). Coinfection outcome in an opportunistic pathogen depends on the inter-strain interactions. BMC Evol. Biol..

[19] Kolton M., Sela N., Elad Y., Cytryn E. (2013). Comparative Genomic Analysis Indicates that Niche Adaptation of Terrestrial *Flavobacteria* Is Strongly Linked to Plant Glycan Metabolism. PLoS ONE.

[20] Nakayama H., Tanaka K., Teramura N., Hattori S. (2016). Expression of collagenase in Flavobacterium psychrophilum isolated from cold-water disease-affected ayu (Plecoglossus altivelis). Biosci. Biotechnol. Biochem..

[21] Kharade S.S., McBride M.J. (2014). Flavobacterium johnsoniae chitinase ChiA is required for chitin utilization and is secreted by the type IX secretion system. J. Bacteriol..

[22] Fuqua C., Parsek M.R., Greenberg E.P. (2001). Regulation of gene expression by cell-to-cell communication: Acyl-homoserine lactone quorum sensing. Annu. Rev. Genet..

[23] Singh V.K., Mishra A., Jha B. (2017). Anti-quorum sensing and anti-biofilm activity of Delftia tsuruhatensis extract by attenuating the quorum sensing-controlled virulence factor production in Pseudomonas aeruginosa. Front. Cell. Infect. Microbiol..

[24] Miller M.B., Bassler B.L. (2001). Quorum sensing in bacteria. Annu. Rev. Microbiol..

[25] Bankevich A., Nurk S., Antipov D., Gurevich A.A., Dvorkin M., Kulikov A.S., Lesin V.M., Nikolenko S.I., Pham S., Prjibelski A.D. (2012). SPAdes: A new genome assembly algorithm and its applications to single-cell sequencing. J. Comput. Biol..

[26] Kolmogorov M., Armstrong J., Raney B.J., Streeter I., Dunn M., Yang F., Odom D., Flicek P., Keane T.M., Thybert D. (2018). Chromosome assembly of large and complex genomes using multiple references. Genome Res..

[27] Waterhouse R.M., Seppey M., Simao F.A., Manni M., Ioannidis P., Klioutchnikov G., Kriventseva E.V., Zdobnov E.M. (2018). BUSCO applications from quality assessments to gene prediction and phylogenomics. Mol. Biol. Evol..

[28] Haack F.S., Poehlein A., Kröger C., Voigt C.A., Piepenbring M., Bode H.B., Daniel R., Schäfer W., Streit W.R. (2016). Molecular Keys to the Janthinobacterium and Duganella spp. Interaction with the Plant Pathogen Fusarium graminearum. Front. Microbiol..

[29] An W., Guo F., Song Y., Gao N., Bai S., Dai J., Wei H., Zhang L., Yu D., Xia M. (2016). Comparative genomics analyses on EPS biosynthesis genes required for floc formation of Zoogloea resiniphila and other activated sludge bacteria. Water Res..

[30] Page A.J., Cummins C.A., Hunt M., Wong V.K., Reuter S., Holden M.T.G., Fookes M., Falush D., Keane J.A., Parkhill J. (2015). Roary: Rapid large-scale prokaryote pan genome analysis. Bioinformatics.

[31] Terrapon N., Lombard V., Drula É., Lapébie P., Al-Masaudi S., Gilbert H.J., Henrissat B. (2018). PULDB: The expanded database of Polysaccharide Utilization Loci. Nucleic Acids Res..

[32] Basson A., Flemming L.A., Chenia H.Y. (2008). Evaluation of adherence, hydrophobicity, aggregation, and biofilm development of Flavobacterium johnsoniae-like isolates. Microb. Ecol..

[33] Lee J., Cho D.H., Ramanan R., Kim B.H., Oh H.M., Kim H.S. (2013). Microalgae-associated bacteria play a key role in the flocculation of Chlorella vulgaris. Bioresour. Technol..

[34] Alexander B.E., Mueller B., Vermeij M.J.A., van der Geest H.H.G., de Goeij J.M. (2015). Biofouling of inlet pipes affects water quality in running seawater aquaria and compromises sponge cell proliferation. PeerJ.

[35] Silverman J.M., Brunet Y.R., Cascales E., Mougous J.D. (2012). Structure and regulation of the type VI secretion system. Annu. Rev. Microbiol..

[36] Wang J., Brodmann M., Basler M. (2019). Assembly and subcellular localization of bacterial type VI secretion systems. Annu. Rev. Microbiol..

[37] Larsbrink J., Zhu Y., Kharade S.S., Kwiatkowski K.J., Eijsink V.G.H., Koropatkin N.M., McBride M.J., Pope P.B. (2016). A polysaccharide utilization locus from Flavobacterium johnsoniae enables conversion of recalcitrant chitin. Biotechnol. Biofuels.

[38] McBride M.J., Xie G., Martens E.C., Lapidus A., Henrissat B., Rhodes R.G., Goltsman E., Wang W., Xu J., Hunnicutt D.W. (2009). Novel features of the polysaccharide-digesting gliding bacterium Flavobacterium johnsoniae as revealed by genome sequence analysis. Appl. Environ. Microbiol..

[39] Overbeek R., Olson R., Pusch G.D., Olsen G.J., Davis J.J., Disz T., Edwards R.A., Gerdes S., Parrello B., Shukla M. (2014). The SEED and the Rapid Annotation of microbial genomes using Subsystems Technology (RAST). Nucleic Acids Res..

[40] Dieser M., Smith H.J., Ramaraj T., Foreman C.M. (2019). Janthinobacterium CG23_2: Comparative Genome Analysis Reveals Enhanced Environmental Sensing and Transcriptional Regulation for Adaptation to Life in an Antarctic Supraglacial Stream. Microorganisms.

[41] Yang M., Lu D., Qin B., Liu Q., Zhao Y., Liu H., Ma J. (2018). Highly efficient nitrogen removal of a coldness-resistant and low nutrient needed bacterium, Janthinobacterium sp. M-11. Bioresour. Technol..

[42] Schloss P.D., Allen H.K., Klimowicz A.K., Mlot C., Gross J.A., Savengsuksa S., McEllin J., Clardy J., Ruess R.W., Handelsman J. (2010). Psychrotrophic Strain of *Janthinobacterium lividum* from a Cold Alaskan Soil Produces Prodigiosin. DNA Cell Biol..

[43] Gong X., Skrivergaard S., Korsgaard B.S., Schreiber L., Marshall I.P.G., Finster K., Schramm A. (2017). High quality draft genome sequence of Janthinobacterium psychrotolerans sp. nov., isolated from a frozen freshwater pond. Stand. Genom. Sci..

[44] Tezuka Y. (1969). Cation-dependent flocculation in a Flavobacterium species predominant in activated sludge. Appl. Microbiol..

[45] Park M., Ryu S.H., Vu T.H.T., Ro H.S., Yun P.Y., Jeon C.O. (2007). Flavobacterium defluvii sp. nov., isolated from activated sludge. Int. J. Syst. Evol. Microbiol..

[46] Hantula J., Bamford D.H. (1991). The efficiency of the protein-dependent flocculation of Flavobacterium sp. is sensitive to the composition of growth medium. Appl. Microbiol. Biotechnol..

[47] Tatusova T., Dicuccio M., Badretdin A., Chetvernin V., Nawrocki E.P., Zaslavsky L., Lomsadze A., Pruitt K.D., Borodovsky M., Ostell J. (2016). NCBI prokaryotic genome annotation pipeline. Nucleic Acids Res..

[48] Asnicar F., Thomas A.M., Beghini F., Mengoni C., Manara S., Manghi P., Zhu Q., Bolzan M., Cumbo F., May U. (2020). Precise phylogenetic analysis of microbial isolates and genomes from metagenomes using PhyloPhlAn 3.0. Nat. Commun..

[49] Li J., Yao Y., Xu H.H., Hao L., Deng Z., Rajakumar K., Ou H.Y. (2015). SecReT6: A web-based resource for type VI secretion systems found in bacteria. Environ. Microbiol..

[50] Okonechnikov K., Golosova O., Fursov M., Varlamov A., Vaskin Y., Efremov I., German Grehov O.G., Kandrov D., Rasputin K., Syabro M. (2012). Unipro UGENE: A unified bioinformatics toolkit. Bioinformatics.

[51] Terrapon N., Lombard V., Gilbert H.J., Henrissat B. (2015). Automatic prediction of polysaccharide utilization loci in Bacteroidetes species. Bioinformatics.

