# Supplementary material for: Cooperative Interaction of Janthinobacterium sp. SLB01 and Flavobacterium sp. SLB02 in the Diseased Sponge Lubomirskia baicalensis
Source: Int J Mol Sci. 2020 Oct 30;21(21):8128. doi: 10.3390/ijms21218128 (PMC7662799; doi:10.3390/ijms21218128)
Supplement: Supplementary file 1 [file ijms-21-08128-s001.zip › Table S1a. Strains Janthinobacterium v1.docx]

**Table S1a.** List of *Janthinobacterium* strains analyzed in this study

| Strain | Isolation source | Genome size, Mbases | CDS count | GenBank accession number |
| --- | --- | --- | --- | --- |
| ***Janthinobacterium* sp. SLB01** | **Diseased sponge, Lake Baikal, Russia** | **6.47** | **5459** | **VZAB01000000** |
| *Janthinobacterium* *lividum* PAMC 25724 | Alpine glacier cryoconite, Korea | 4.98 | 4191 | AHHB01000000 |
| *Janthinobacterium* *lividum* strain RIT308 | Shrub willow (salix) grown in geneva, USA | 6.21 | 5436 | JFYR01000000 |
| *Janthinobacterium* *lividum* strain MTR | Cajon del Maipo, Santiago Metropolitan Region, Chile | 6.54 | 5756 | JRRH01000000 |
| *Janthinobacterium* *lividum* strain NFR18 | n/a | 6.30 | 5511 | FPKH01000000 |
| *Janthinobacterium* sp. RA13 | Lake Washington Sediment, USA | 6.42 | 5619 | JQNP00000000 |
| *Janthinobacterium* sp. HH102 | Water rainwater-tap Botanic Garden, Hamburg, Germany | 6.65 | 5977 | LRHZ00000000 |
| *Janthinobacterium* sp. MP5059B | Soil, Germany | 6.46 | 5661 | LRHX00000000 |
| *Janthinobacterium* sp. BJB301 | Hudson Valley Watershed, USA | 6.39 | 5592 | PDZJ00000000 |
