# Supplementary material for: Cooperative Interaction of Janthinobacterium sp. SLB01 and Flavobacterium sp. SLB02 in the Diseased Sponge Lubomirskia baicalensis
Source: Int J Mol Sci. 2020 Oct 30;21(21):8128. doi: 10.3390/ijms21218128 (PMC7662799; doi:10.3390/ijms21218128)
Supplement: Supplementary file 1 [file ijms-21-08128-s001.zip › Table S1b. Strains Flavobacterium v2.docx]

**Table S1b.** List of *Flavobacterium* strains analyzed in this study

| Strain | Isolation source | Niche (aquatic/ terrestrial) | Genome size, Mbases | CDS count | GenBank accession number |
| --- | --- | --- | --- | --- | --- |
| ***Flavobacterium* sp. SLB02** | **Diseased sponge, Lake Baikal, Russia** | **aquatic** | **6.36** | **4828** | **CP045928** |
| *Flavobacterium* *johnsoniae* UW101 | Soil; UK | terrestrial | 6.10 | 5091 | NC_009441 |
| *Flavobacterium* sp. F52 | Rhizosphere of pepper; Israel | terrestrial | 5.34 | 4497 | AKZQ00000000 |
| *Flavobacterium* sp. URHB0058 | Forest soil; North America | terrestrial | 5.26 | 4408 | AUEU00000000 |
| *Flavobacterium* sp. CF136 | Tree rhizosphere; USA | terrestrial | 5.10 | 4166 | AKJZ00000000 |
| *Flavobacterium* sp. B17 | Rice shoot; Japan | terrestrial | 4.17 | 3439 | BACY00000000 |
| *Flavobacterium* *soli* DSM 19725 | Soil samples; South Korea | terrestrial | 4.00 | 3512 | AUGO00000000 |
| *Flavobacterium* *antarcticum* DSM 19726 | Soil sample; Antarctica | terrestrial | 3.08 | 2720 | ATTM00000000 |
| *Flavobacterium* sp. WG21 | Wintergreen Lake; USA | aquatic | 5.20 | 4131 | AMYW00000000 |
| *Flavobacterium* *rivuli* DSM 21788 | Hard water stream; Germany | aquatic | 4.49 | 3869 | ARKJ00000000 |
| *Flavobacterium* sp. ACAM 123 | Burton Lake; Antarctic lake | aquatic | 3.96 | 3161 | AJXL00000000 |
| *Flavobacterium* *frigoris* PS1 | Sea ice, diatom layer; Antarctic lake | aquatic | 3.93 | 3318 | AHKF00000000 |
| *Flavobacterium* *branchiophilum* FL-15 | Diseased sheatfish; Hungary | aquatic | 3.56 | 3935 | NC_016001 |
| *Flavobacterium* *columnare* ATCC 49512 | Skin lesion of trout fry; France | aquatic | 3.16 | 2616 | NC_016510 |
| *Flavobacterium* *indicum* GPTSA100-9 | Warm spring water; India | aquatic | 2.99 | 2679 | NC_017025 |
| *Flavobacterium* *psychrophilum* JIP02/86 | Kidney of rainbow trout; France | aquatic | 2.86 | 2294 | NC_009613 |
