# Supplementary material for: Cooperative Interaction of Janthinobacterium sp. SLB01 and Flavobacterium sp. SLB02 in the Diseased Sponge Lubomirskia baicalensis
Source: Int J Mol Sci. 2020 Oct 30;21(21):8128. doi: 10.3390/ijms21218128 (PMC7662799; doi:10.3390/ijms21218128)
Supplement: Supplementary file 1 [file ijms-21-08128-s001.zip › Table S1c. Phylogenetic software settings.docx]

**Table S1c.** List and parameters of software used to build phylogenetic trees

| Software name | Version | Parameters list |
| --- | --- | --- |
| BLASTp | 2.9.0+ | --quiet --threads 1 --outfmt 6 --more-sensitive --id 50 --max-hsps 35 -k 0 |
| diamond | 0.9.32 | (defaults) |
| MAFFT | 7.455 | --quiet --anysymbol --thread 1 --auto |
| FastTree | 2.1.10 | -quiet -mlacc 2 -slownni -spr 4 -fastest -mlnni 4 -no2nd -lg |
| RAxML | 8.2.11 | -p 1989 -m PROTCATLG -T 8 |
| trimal | 1.4.rev15 | -gappyout |
