# Supplementary material for: Cooperative Interaction of Janthinobacterium sp. SLB01 and Flavobacterium sp. SLB02 in the Diseased Sponge Lubomirskia baicalensis
Source: Int J Mol Sci. 2020 Oct 30;21(21):8128. doi: 10.3390/ijms21218128 (PMC7662799; doi:10.3390/ijms21218128)
Supplement: Supplementary file 1 [file ijms-21-08128-s001.zip › Table S2 Violacein production genes localization into Janthinobacterium sp. SLB01 genome.docx]

**Table S2.** Violacein production genes localization into *Janthinobacterium* sp. SLB01 genome

| Gene name | Locus tag | begin | end | strand | Length, bp |
| --- | --- | --- | --- | --- | --- |
| vioA | F3B38_RS17235 | 1353472 | 1354779 | + | 1308 |
| vioB | F3B38_RS17240 | 1354776 | 1357796 | + | 3021 |
| vioC | F3B38_RS17245 | 1357798 | 1359087 | + | 1290 |
| vioD | F3B38_RS17250 | 1359087 | 1360205 | + | 1119 |
| vioE | F3B38_RS17255 | 1360216 | 1360216 | + | 582 |
