# Supplementary material for: Cooperative Interaction of Janthinobacterium sp. SLB01 and Flavobacterium sp. SLB02 in the Diseased Sponge Lubomirskia baicalensis
Source: Int J Mol Sci. 2020 Oct 30;21(21):8128. doi: 10.3390/ijms21218128 (PMC7662799; doi:10.3390/ijms21218128)
Supplement: Supplementary file 1 [file ijms-21-08128-s001.zip › Table S3 T6SS genes.docx]

**Table S3.** Type VI secretion system loci into *Janthinobacterium* sp. SLB01 genome

| Compo-nent | Locus tag | begin | end | strand | Length, bp | Annotation |
| --- | --- | --- | --- | --- | --- | --- |
| Hcp | F3B38_RS13910 | 597756 | 598325 | – | 570 | Hcp1 family type VI secretion system effector |
| VgrG | F3B38_RS07010 | 1749246 | 1752041 | + | 2796 | type VI secretion system tip protein VgrG |
|  | F3B38_RS13765 | 558656 | 561964 | + | 3309 |  |
|  | F3B38_RS13780 | 566569 | 569388 | + | 2820 |  |
|  | F3B38_RS19390 | 1878802 | 1881399 | + | 2598 |  |
|  | F3B38_RS23050 | 2696791 | 2698351 | – | 1561 |  |
| ClpV | F3B38_RS20615 | 2147585 | 2148961 | – | 1377 | DNA repair protein RadA |
| TssB | F3B38_RS21310 | 2290983 | 2291492 | – | 510 | type VI secretion system contractile sheath small subunit |
| TssC | F3B38_RS21305 | 2289482 | 2290981 | – | 1500 | type VI secretion system contractile sheath large subunit |
| TssJ | F3B38_RS21320 | 2292405 | 2293004 | + | 600 | type VI secretion system lipoprotein TssJ |
| TssL | F3B38_RS21330 | 2294422 | 2295207 | + | 786 | DotU family type IV/VI secretion system protein |
| TssM | F3B38_RS21345 | 2297101 | 2300949 | – | 3849 | type VI secretion system membrane subunit TssM |
